# Natural NADH and FAD Autofluorescence as Label-Free Biomarkers for Discriminating Subtypes and Functional States of Immune Cells

**DOI:** 10.3390/ijms23042338

**Published:** 2022-02-20

**Authors:** Sarah Lemire, Oana-Maria Thoma, Lucas Kreiss, Simon Völkl, Oliver Friedrich, Markus F. Neurath, Sebastian Schürmann, Maximilian J. Waldner

**Affiliations:** 1 Department of Internal Medicine 1, University Hospital Erlangen, Friedrich-Alexander-Universität Erlangen-Nürnberg (FAU), 91052 Erlangen, Germany; oana-maria.thoma@uk-erlangen.de (O.-M.T.); lucas.kreiss@fau.de (L.K.); markus.neurath@uk-erlangen.de (M.F.N.); 2 Deutsches Zentrum Immuntherapie, University Hospital Erlangen, Friedrich-Alexander-Universität Erlangen-Nürnberg (FAU), 91054 Erlangen, Germany; 3 Institute of Medical Biotechnology, Friedrich-Alexander-Universität Erlangen-Nürnberg (FAU), 91052 Erlangen, Germany; oliver.friedrich@fau.de (O.F.); sebastian.schuermann@fau.de (S.S.); 4 Department of Internal Medicine 5, Haematology and Oncology, University Hospital Erlangen, Friedrich-Alexander-Universität Erlangen-Nürnberg (FAU), 91054 Erlangen, Germany; simon.voelkl@uk-erlangen.de; 5 Erlangen Graduate School in Advanced Optical Technologies, Friedrich-Alexander-Universität Erlangen-Nürnberg (FAU), 91052 Erlangen, Germany

**Keywords:** inflammatory bowel diseases, cell autofluorescence, immune cells, NADH, FAD, flow cytometry, multiphoton microscopy

## Abstract

Immune cell activity is a major factor for disease progression in inflammatory bowel diseases (IBD). Classifying the type and functional state of immune cells is therefore crucial in clinical diagnostics of IBD. Label-free optical technologies exploiting NADH and FAD autofluorescence, such as multiphoton microscopy, have been used to describe tissue morphology in healthy and inflamed colon samples. Nevertheless, a strategy for the identification of single immune cell subtypes within the tissue is yet to be developed. This work aims to initiate an understanding of autofluorescence changes depending on immune cell type and activation state. For this, NADH and FAD autofluorescence signals of different murine immune cell subtypes under native conditions, as well as upon in vitro stimulation and cell death, have been evaluated. Autofluorescence was assessed using flow cytometry and multiphoton microscopy. Our results reveal significantly increased NADH and FAD signals in innate immune cells compared to adaptive immune cells. This allowed identification of relative amounts of neutrophils and CD4+ T cells in mixed cell suspensions, by using NADH signals as a differentiation marker. Furthermore, in vitro stimulation significantly increased NADH and FAD autofluorescence in adaptive immune cells and macrophages. Cell death induced a significant drop in NADH autofluorescence, while FAD signals were hardly affected. Taken together, these results demonstrate the value of autofluorescence as a tool to characterize immune cells in different functional states, paving the way to the label-free clinical classification of IBD in the future.

## 1. Introduction

Immune cells are highly important for maintaining health and homeostasis and play a key role in the pathogenesis of inflammatory bowel diseases (IBD). Specific T-cell subtypes have been directly linked to disease progression in IBD. For example, helper T cells (Th1, Th2 and Th17 cells) trigger gut inflammation [1,2], while regulatory T cells (Treg) help to prevent the inflammation-related destruction of the mucosa [1]. Similarly, adaptive immune cells, such as macrophages and neutrophils, are involved in the pathogenesis of mucosal inflammation. For instance, pro-inflammatory M1 macrophages can support inflammation through the release of pro-inflammatory cytokines, whereas M2 macrophages have anti-inflammatory properties [3]. Neutrophil infiltration in the gut mucosa has been linked to high disease activity in ulcerative colitis (UC), while a lack of neutrophils leads to an uncontrolled invasion of bacteria that promote inflammation in Crohn’s disease (CD) [4]. Understanding how immune cells modulate IBD is an important area of research. Therefore, a specific identification and evaluation of immune cell subtypes, activation states, proliferation and cell death could reveal important information on the current state and future course of disease in IBD. 

Optical technologies, such as multiphoton microscopy (MPM), have lately received increasing interest as label-free options to investigate the native tissue morphology of healthy and inflamed colon based on autofluorescence [5,6]. Autofluorescence is a cell property that depends on the presence of endogenous fluorescent molecules, such as NADH and FAD [7]. Using this natural autofluorescence is highly advantageous, as label-free imaging strategies do not affect the state of individual cells. While MPM allows for the identification of intrastromal cells in colitis models [5], a clear classification of infiltrating immune cell subtypes and functional states is not yet available. Therefore, laying a foundation for understanding how autofluorescence signals differ in individual types of immune cells and their activation status is relevant for possible clinical applications in IBD patients.

This study analysed NADH and FAD signals of murine immune cells in different functional states using flow cytometry and MPM. Flow cytometry, on the one hand, is a broadly used method, able to analyse high numbers of cells in a short period of time [8]. Although usually used to characterize cells with the help of fluorescent markers, it has also shown its potential in detecting autofluorescent signals in different types of cells [9,10,11]. MPM is a promising optical imaging technique exploiting the non-linear excitation of fluorescent molecules including NADH and FAD [12]. This allows successful label-free evaluation of 3D tissue morphology, as we have described in previous studies [5]. The specific characteristics of NADH and FAD autofluorescence in single immune cell types and under different functional conditions, however, remain to be described. Therefore, the purpose of this study was to evaluate autofluorescence profiles in various types of innate and adaptive immune cells and how these are affected by different functional states. This might be a first step towards a label-free classification of infiltrating immune cells in the clinical diagnostics of IBD.

## 2. Results 

### 2.1. Adaptive and Innate Immune Cells Show Different NADH and FAD Signals in Flow Cytometry

**Figure 1:**
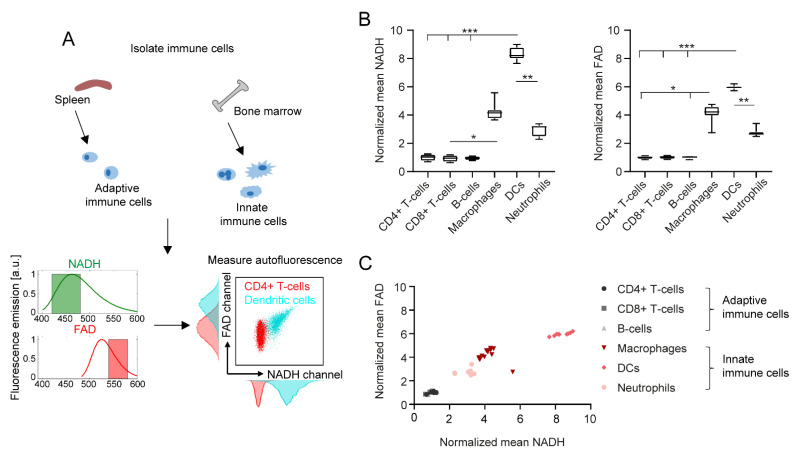
Immune cell subtypes can be differentiated by NADH and FAD autofluorescence signals in flow cytometry. (**A**) Schematic representation of immune cell isolation from murine spleen and bone marrow, and exemplary autofluorescence analysis in flow cytometry. (**B**) NADH and FAD signal mean values of different immune cell subtypes, normalized to the mean values of alive, unstimulated CD4+ T cells. For statistical analysis, a Kruskal–Wallis test with subsequent Dunn’s multiple comparisons test was used (* *p* < 0.05, ** *p* < 0.01, *** *p* < 0.001). At least four samples per group were analysed. Each sample reflects either cells isolated from one mouse (CD4+ T cells, CD8+ T cells, B cells, neutrophils) or cells cultured in one culture dish (macrophages, dendritic cells). (**C**) Comparison of normalized mean NADH and FAD signals in different immune cell types.

In Figure 1A, we schematically describe the workflow from immune cell isolation to autofluorescence measurements and NADH/FAD detection by flow cytometry. As explained in Section 4, the filters used for label-free flow cytometry of immune cells mainly collected NADH/FAD autofluorescence signals; however, autofluorescence signals from other endogenous fluorophores cannot be completely avoided. The respective intensities of NADH and FAD signals among different immune cell types (e.g., CD4+ T cells and dendritic cells) are illustrated in the exemplary NADH/FAD dot plots and histograms. Similar histograms for further immune cell subtypes are presented in Supplementary Figure S1. Quantification of NADH and FAD fluorescence revealed a considerable increase in both fluorescent molecules in innate immune cells compared to adaptive immune cells (Figure 1B). 

Overall, dendritic cells showed the strongest NADH and FAD signals of all analysed cell types (eight-fold increase in NADH and six-fold increase in FAD compared to adaptive immune cells). Furthermore, NADH and FAD signals of dendritic cells were also significantly different to those of neutrophils (2.8-fold increase in NADH, 2.1-fold increase in FAD). The autofluorescence signals among CD4+ T cells, CD8+ T cells and B cells did not show any relevant differences. Interestingly, changes in NADH and FAD signals correlated with each other in the different cell types, as shown in Figure 1C. 

### 2.2. In Vitro Stimulation Increases NADH and FAD Signals in Flow Cytometry

**Figure 2:**
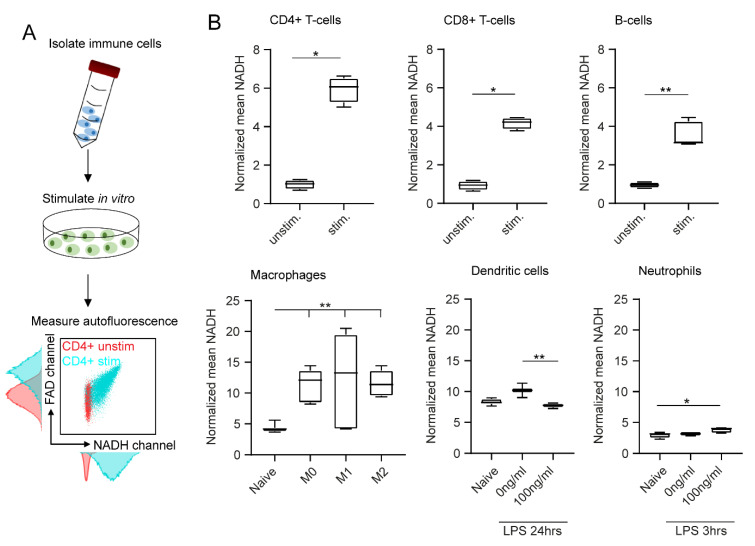
In vitro stimulation affects immune cell autofluorescence in flow cytometry. (**A**) After in vitro stimulation, immune cell autofluorescence was assessed by flow cytometry. (**B**) Boxplots showing NADH autofluorescence of adaptive and innate immune cells before and after in vitro stimulation. Statistical significance was analysed with a Mann–Whitney test for comparing two independent groups. For three independent groups, a Kruskal–Wallis test with subsequent Dunn’s multiple comparisons test was used (* *p* < 0.05, ** *p* < 0.01). At least three samples per group were analysed. Each sample reflects either cells isolated from one mouse (unstimulated CD4+ T cells, CD8+ T cells, B cells, neutrophils) or cells cultured in one culture dish (stimulated CD4+ T cells, CD8+ T cells, B cells, neutrophils; unstimulated and stimulated macrophages, dendritic cells).

In addition to the type of cell, we identified in vitro stimulation as another important determinant of cell autofluorescence. As described in Figure 2A, we performed in vitro stimulation of all six immune cell types and analysed their NADH and FAD signals with flow cytometry. Histograms and dot plots of unstimulated and stimulated CD4+ T cells are shown exemplarily and illustrate a significant shift in NADH and FAD autofluorescence after stimulation. For instance, NADH increased six-fold in stimulated CD4+ T cells compared to unstimulated CD4+ T cells (Figure 2B). 

Significant differences were also observed when comparing unstimulated and stimulated CD8+ T cells (4.5-fold increase in NADH) and unstimulated and stimulated B cells (3.7-fold increase in NADH). Polarized macrophages showed similar results: all three polarization groups displayed significantly increased NADH signals compared to the unstimulated cells (2.9-fold increase in NADH). Interestingly, the type of polarization had no major effect on autofluorescence signals. Dendritic cells and neutrophils were only weakly affected by in vitro stimulation (Figure 2B). FAD values displayed similar results in all cases (data not shown). In conclusion, we observed clear effects of in vitro stimulation on autofluorescence signals of adaptive immune cells and macrophages. 

### 2.3. NADH Autofluorescence Is Decreased in Dead Cells

**Figure 3:**
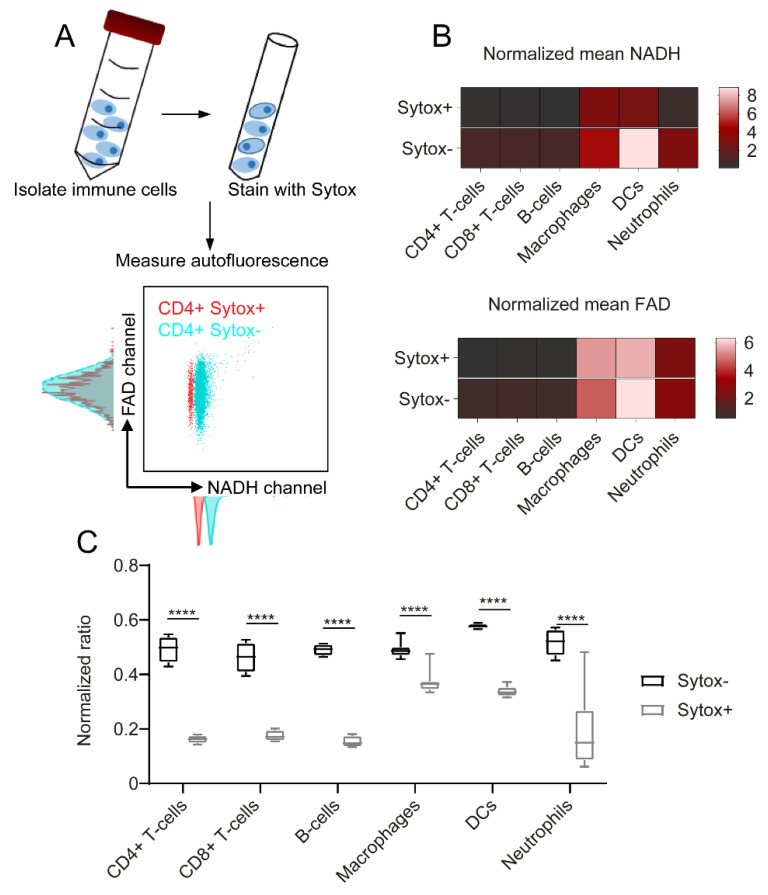
NADH autofluorescence decrease upon cell death as shown by flow cytometry. (**A**) To identify dead cells, the samples were stained with Sytox Red after isolation. Autofluorescence signals were measured using flow cytometry. (**B**) NADH and FAD signals of dead (Sytox+) and alive (Sytox−) cells, illustrated in a heat map. (**C**) Redox ratios calculated as R = NADH/(NADH + FAD). For statistical analysis, two-way ANOVA with Sidak’s multiple comparisons test was used (**** *p* < 0.0001). At least four samples per group were analysed. Each sample reflects either cells isolated from one mouse (CD4+ T cells, CD8+ T cells, B cells, neutrophils) or cells cultured in one culture dish (macrophages, dendritic cells).

To analyse the effect of cell death on immune cell autofluorescence, cells were stained with the cell death marker Sytox Red. Figure 3A exemplarily illustrates the effect of cell death on unstimulated CD4+ T cells: while NADH autofluorescence was shifted to decreased values in the Sytox+ dead cell population compared to the Sytox- alive cells, FAD signals remained constant. Heat maps in Figure 3B describe similar patterns for all the other immune cell types and show that cell death only had an impact on NADH autofluorescence signals, whereas FAD signals were not, or only slightly, affected. To illustrate this finding, we calculated the optical redox ratio as R = NADH/(NADH + FAD) and compared the results for dead and alive cells. As expected, this ratio was significantly decreased in Sytox+ dead cells due to the drop in NADH autofluorescence (Figure 3C). The strongest differences were observed within adaptive immune cell populations (three-fold decrease in ratio) and dendritic cells (1.7-fold decrease in ratio); macrophages showed the smallest decrease in the NADH autofluorescence of dead cells (1.3-fold decrease in ratio). Taken together, these findings show a considerable impact of cell death on NADH autofluorescence, while FAD signals were not significantly affected.

### 2.4. NADH and FAD Signals of Immune Cells Can Be Analysed with MPM

**Figure 4:**
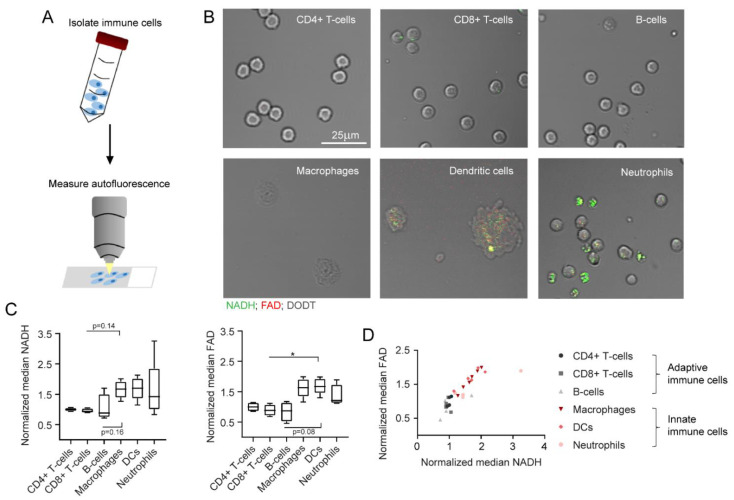
NADH and FAD autofluorescence changes as measured in multiphoton microscopy. (**A**) Schematic representation of MPM measurements after immune cell isolation from murine spleen and bone marrow. (**B**) Exemplary images of different immune cell types, as measured in MPM. NADH: green, FAD: red, DODT: grey (scalebar: 25 µm). (**C**) Median NADH and FAD signals of different immune cells, normalized to NADH and FAD signals of alive, unstimulated CD4+ T cells. For statistical analysis, a Kruskal–Wallis test with Dunn’s multiple comparisons test was used (* *p* < 0.05). At least four samples per group were analysed. Each sample includes at least 30–500 cells and reflects either cells isolated from one mouse (CD4+ T cells, CD8+ T cells, B cells, neutrophils) or cells cultured in one culture dish (macrophages, dendritic cells). (**D**) Comparison of normalized median NADH and FAD signals.

In the second part of our study, all immune cell subtypes were analysed with MPM (Figure 4A). According to the filter setup described in Section 4, NADH and FAD predominantly contributed to the autofluorescence signals measured in this study. Nevertheless, other autofluorescent molecules might also influence cell autofluorescence to a small extent. Figure 4B depicts the NADH and FAD signals of all six immune cell subtypes as shown by MPM. NADH signals are illustrated in green, FAD signals are shown in red and signals from the DODT channel are displayed in grey. The immune cell subtypes morphologically differed in shape and size, but also revealed differences in the strength of NADH and FAD signals. Innate immune cells showed stronger autofluorescence, whereas the signal in adaptive immune cells was generally weak. Quantification of the data displayed similar trends to what we observed with flow cytometry: innate immune cells had higher NADH (1.6-fold increase) and FAD (1.7-fold increase) autofluorescence signals in comparison to adaptive immune cells (Figure 4C). Furthermore, signal intensities of NADH correlated with FAD signal intensities for all cell types (Figure 4D). 

### 2.5. NADH and FAD Signals Increase upon In Vitro Stimulation as Measured by MPM

**Figure 5:**
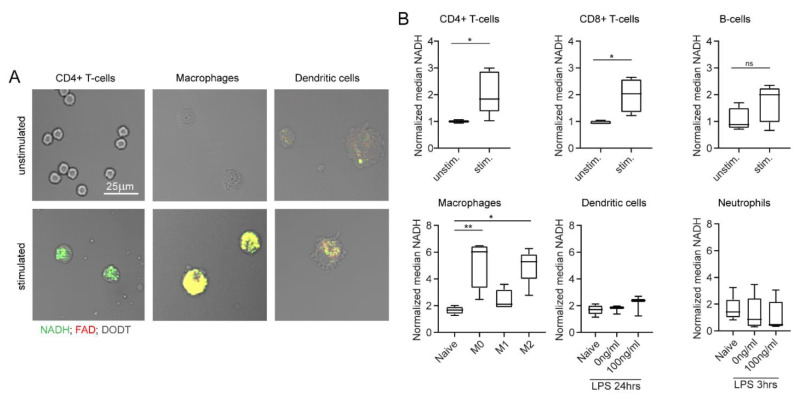
In vitro stimulation changes NADH and FAD autofluorescence signals, as measured by multiphoton microscopy. (**A**) Example images of unstimulated and stimulated CD4+ T cells, macrophages, and dendritic cells. NADH: green, FAD: red, DODT: grey (scalebar: 25 µm). (**B**) NADH autofluorescence signals of adaptive and innate immune cells after in vitro stimulation. Statistical significance was analysed with a Mann–Whitney test for comparing two independent groups. For three independent groups, a Kruskal–Wallis test with subsequent Dunn’s multiple comparisons test was used (* *p* < 0.05, ** *p* < 0.01). At least three samples per group were analysed. Each sample includes at least 30–500 cells and reflects either cells isolated from one mouse (unstimulated CD4+ T cells, CD8+ T cells, B cells, neutrophils) or cells cultured in one culture dish (stimulated CD4+ T cells, CD8+ T cells, B cells, neutrophils; unstimulated and stimulated macrophages, dendritic cells).

Additionally, we analysed the effect of in vitro stimulation on immune cells in MPM. For this, autofluorescence signals of unstimulated and stimulated cells were compared, analogous to what we have described for flow cytometry measurements. The increase in NADH and FAD signals was especially visible in CD4+ T cells and macrophages (Figure 5A). This visual impression was confirmed by quantification in Figure 5B: we observed a significant increase in NADH signals in stimulated adaptive immune cells compared to unstimulated adaptive immune cells (1.9-fold increase in NADH). Furthermore, macrophage polarization induced a significant NADH increase (2.5-fold increase in NADH compared to unpolarized macrophages). No relevant effects of in vitro stimulation were observed among dendritic cells and neutrophils. FAD values showed similar results in all cases (data not shown). Overall, these findings correlate with our results from flow cytometry measurements and demonstrate the feasibility of assessing single cell autofluorescence with MPM.

### 2.6. Autofluorescence as a Tool to Distinguish Cell Types in a Mixed Cell Suspension

**Figure 6:**
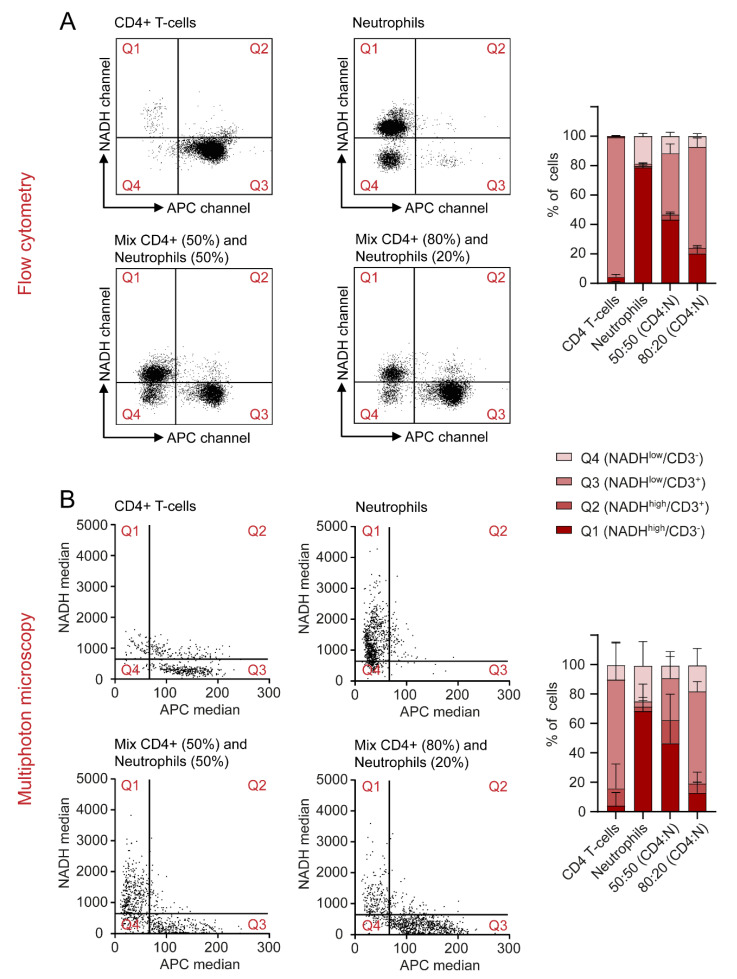
NADH autofluorescence signals may be used to differentiate between cell types in a mixed sample in flow cytometry and multiphoton microscopy. (**A**,**B**) Cells were divided into four quadrants, defined by high and low NADH and APC values. CD4+ T cells and neutrophils were analysed on their own and in mixed samples and the percentages of cells found in each quadrant were represented for both flow cytometry and MPM.

The final aim of this study was to detect relative amounts of two different immune cell types in a mixed suspension by quantifying autofluorescence signatures by flow cytometry and MPM. CD4+ T cells and neutrophils were chosen as the most suitable mixture, since they differ regarding their NADH signals, while showing similar morphology and size. As described in Section 4, NADH and APC values of single cells were measured for all four conditions of cell mixes. APC-labelled α-CD3 was used as a marker to identify CD4+ T cells. Results were visualized and quantified in dot plots for MPM and flow cytometry and dot plots were divided into four quadrants defined by high and low NADH and APC values. Exemplary dot plots are presented in Figure 6A,B. Furthermore, we calculated the percentage of cells found in each quadrant. In the samples that only contained CD4+ T cells, 94.88% of cells in flow cytometry and 73.98% of cells in MPM displayed low NADH and high APC. For neutrophils alone, most cells were defined by high NADH and low APC (78.7% flow cytometry, 68.37% MPM). In the mixed samples, both conditions occurred according to the mixing ratio in the sample. Interestingly, a cell population displaying low NADH and low APC was found in the samples containing isolated neutrophils. These cells were confirmed as B cells in flow cytometry (data not shown) and can hardly be avoided due to the isolation protocol used for neutrophils. Taken together, this experiment showed that NADH autofluorescence values may be used as a potential identification tool for different immune cell subtypes, without the need for additional staining or morphological characterization. 

## 3. Discussion

This study was designed to evaluate label-free, optical differentiation of immune cell subtypes and functional states without relying on exogenous and artificial markers. This work is relevant to many inflammatory diseases, such as IBD, since it could allow semi-specific classification of immune cell groups based on native tissue samples. In the context of IBD, MPM has already been used to describe the 3D morphology of the gut mucosa in untreated tissue biopsies or during in vivo endomicroscopy. Thereby, it was suggested that increased numbers of stromal cells in different colitis models represent infiltrating immune cells [5]. Yet, a clear identification of single immune cell types and functional states within this tissue remained difficult, even though this knowledge can be valuable in the clinical diagnostics of IBD [3,13,14].

In unstimulated immune cells, we observed an increase in NADH and FAD signals in innate immune cells compared to adaptive immune cells in flow cytometry and MPM. Similar changes were previously described in macrophages and dendritic cells [15]. These findings might be explained by the increased cell granularity in innate immune cells due to high lysosome activity, which can contribute to cell autofluorescence [16,17,18]. Not surprisingly, the activation of immune cells also changes NADH and FAD autofluorescence. This might be the result of increased glycolysis and promotion of oxidative phosphorylation [19,20]. However, not all immune cells showed a change in autofluorescence upon in vitro stimulation. For example, in neutrophils, LPS is not able to directly activate the enzyme NAD(P)H oxidase and can therefore not induce extensive oxidation of NAD(P)H to NAD(P)+ [21,22,23].

Overall, changes in NADH and FAD signals in both steady state and activated immune cells might provide additional information about their behaviour in inflammatory diseases, such as IBD. For example, the recognition of stimulated T cells based on increased NADH and FAD signals may allow the differentiation of healthy and diseased colon tissue in the future. Furthermore, monitoring macrophage activation using the strong increase in NADH and FAD autofluorescence may help to identify early states of gut inflammation, as the activation of macrophages is a crucial step in the initiation of IBD [24]. Similarly, neutrophil infiltration in the gut could be tracked by high autofluorescence signals, as this is an important indicator of acute gut inflammation. 

In this study, we report for the first time that cell death induces a decrease in NADH signals of different immune cell types, while hardly affecting FAD autofluorescence. One important cause of cell death is oxidative stress, which promotes the oxidation of NADH and FADH_2_ to NAD+ and FAD [25]. Since NAD+ is not autofluorescent at the investigated wavelength [26], this explains the strong decrease in NADH signal during cell death. In the context of IBD, immune cell apoptosis plays an essential role in therapeutic strategies. For example, the mechanism of action in drugs, such as azathioprine and anti-TNF antibodies, relies on cell death induction in T cells [27]. Therefore, identification of apoptotic immune cells only based on the NADH/FAD autofluorescence ratio may be used to monitor therapeutic success in IBD patients in the future. 

On the other hand, anti-TNF antibodies, such as infliximab or adalimumab, are not only regulators of T-cell apoptosis, but can also block macrophage activation in the gut mucosa [28]. Therefore, NADH and FAD signals might decrease as an effect of the non-activation of intestinal macrophages. Similar mechanisms can be discussed for other biologicals currently used in the therapy of IBD. Vedolizumab, an α4β7-integrin-specific antibody, inhibits the infiltration or so-called homing of T cells into the gut mucosa, while pro-inflammatory T cell activation can be blocked by ustekinumab, an antibody against IL12 and IL23 [28]. Both substances might lead to a measurable decrease in NADH and FAD signals in the intrastromal cells, as therapy leads to a downregulation of activated T cells. These examples illustrate the potential value of label-free diagnostics to easily assess current therapy strategies in IBD patients. 

Despite this potential, certain limitations of this study must be considered. Although MPM and flow cytometry allow the evaluation of immune cell autofluorescence, flow cytometry usually achieves clearer results. A possible reason for this fact is that flow cytometry was designed for measurements at high throughput, while MPM is usually used as an imaging technology without direct quantification. The number of cells was therefore limited in MPM investigation. Furthermore, we observed that activation and cell type can lead to equivalent changes in autofluorescence. Therefore, autofluorescence signals on their own might not be enough to describe in detail immune cell types and function. A combination of different components in label-free diagnostics, including size, morphology, and granularity of cells should be established in the future.

In conclusion, our findings reveal that cell type, state and function have measurable effects on NADH and FAD autofluorescence signals of isolated murine immune cells in vitro. Such a semi-specific categorization of immune cells could allow the identification of immune cells infiltrating the lamina propria of healthy and inflamed colon tissue in the future. To make autofluorescence, as a clinical parameter, accessible to patients with IBD, application of this research to human samples, including the blood and tissue of IBD patients, is essential. As a long-term goal, the results of this research might lead to the development of automated artificial intelligence systems, able to recognize immune cell type and function based on autofluorescence signals in the diagnostics of IBD patients, paving the way to promising clinical applications.

## 4. Materials and Methods

### 4.1. Adaptive Immune Cell Isolation and Stimulation

This study was prepared and conducted within a period of 14 months between October 2020 and December 2021. 

All immune cell subtypes were obtained from the spleen (CD4+/CD8+ T cells, B cells) or bone marrow (macrophages, dendritic cells, neutrophils) of wildtype C57BL/6 mice. To purify CD4+ and CD8+ T cells, as well as B cells, negative selection of the three different cell types was performed according to the respective isolation kits (Miltenyi Biotec, Bergisch Gladbach, Germany). Purity reached values of >95%. For stimulation, CD4+ and CD8+ T cells were seeded in pre-coated 24-well-plates (α-CD3/α-CD28, 10 µg/mL) and incubated for 5 days. B cells were stimulated with IL4 (10 ng/mL), LPS (5 µg/mL) and α-IgM (10 µg/mL) in 24-well-plates for 3 days.

### 4.2. Maturation and Stimulation of Bone Marrow Derived Cells

Macrophages and dendritic cells were both generated from murine bone marrow progenitor cells, as partly described elsewhere [29,30,31,32]. In short, the femur and tibia were flushed, and cells were resuspended in DMEM/F12 (Anprotec, Bruckberg, Germany) supplemented with 10% FCS (PAN-Biotech, Aidenbach, Germany) and 1% P/S (Sigma-Aldrich, St. Louis, USA). To obtain macrophages, cells were then plated in 6-well-plates and stimulated with M-CSF (20 ng/mL) for 7 days. On day 3, 1 mL of supplemented DMEM/F12 containing M-CSF was added to the cells. On day 7, macrophage polarization was performed: cells were resuspended in 2 mL of DMEM (Gibco, Waltham, MA, USA) supplemented with 10% FCS and 1% P/S and stimulated with either LPS (100 ng/mL) and IFN-γ (50 ng/mL) to obtain M1 macrophages or with IL-4 (20 ng/mL) to obtain M2 macrophages. To generate dendritic cells, bone marrow progenitors were seeded in 6-well-plates and stimulated with IL4 (10 ng/mL) and GM-CSF (15 ng/mL). On day 3, cells were washed and resuspended in 2 mL of supplemented DMEM/F12 containing IL4 and GM-CSF. On day 5, 2 mL of complete DMEM/F12 supplemented with IL4 and GM-CSF were added to the cells. For stimulation, dendritic cells were removed from the plate on day 7 and re-plated for 24 h in a 24-well-plate. LPS (100 ng/mL) was used as a stimulation factor.

### 4.3. Neutrophil Isolation and Stimulation

Neutrophils were isolated from murine bone marrow cells with a density gradient, as described previously [33]. Bones were flushed and cells were resuspended in 2 mL of PBS (Sigma-Aldrich, St. Louis, MO, USA). Three layers of Percoll (Cytiva, Uppsala, Sweden) in different concentrations (78%, 66%, 54%) were prepared and bone marrow cells were carefully layered on top. After centrifugation (1545× *g*, 30 min, 20 °C, no deceleration), the neutrophil layer was collected. Cell purity was >60%. Neutrophil stimulation was performed using LPS (100 ng/mL) in a 24-well-plate for 3 h.

### 4.4. Flow Cytometry

Experiments were performed using a BD LSRFortessa flow cytometer (BD Biosciences, Franklin Lakes, USA) within 4 hours after isolation or collection of the cells. Autofluorescence of all samples was excited with a 355 nm UV-laser source and signals were detected with bandpass filters for NADH (BP 450/50) and FAD (BP 560/40). Additionally, samples were stained with the cell death marker Sytox Red. The cell mixes analysed in Figure 6 were stained with APC-labelled α-CD3. These two markers were chosen specifically as they do not interfere with the emission spectra of NADH and FAD and were excited by a second laser source (633 nm). The emitted signal was collected with a 670/14 bandpass filter. Data were analysed with the FlowJo software (BD Life Sciences, Franklin Lakes, USA). An example of the gating strategy can be found in Supplementary Figure S1. For reasons of simplicity and comparability, a mean value of the NADH and FAD signals was acquired for all samples and normalized to the mean value of alive, unstimulated CD4+ T-cells. These normalized signals are further referred to as NADH and FAD, respectively.

### 4.5. Multiphoton Microscopy

All samples were measured with an upright multiphoton microscope (TriMScope II, LaVision BioTec, Bielefeld, Germany) within 6–8 hrs after isolation or collection of the cells. A 50 µL droplet of cell suspension was placed on a glass slide and cells were allowed to settle for 5 min before covering the slide with a cover slip. Using a 25× water-immersion objective (HC Fluortar 178 L 25×/0.95 W VISIR, Leica microsystems, Wetzlar, Germany), the focal plane was adjusted and autofluorescence was excited with a mode-locked fs-pulsed Ti:Sa laser (Chameleon Vision II, Coherent, Santa Clara, USA) at 810 nm. Two bandpass filters (BP 450/70 and BP 560/40) were used to detect autofluorescence. These two bandpass filters were chosen to collect mainly the emitted light from NADH (BP 450/70) and FAD (BP 560/40). Still, other endogenous fluorophores might also contribute to cell autofluorescence to a small extent. Additionally, the DODT transmission signal, which uses scattering of the excitation laser to visualize 3D morphology of cells, was collected. In every sample, three representative positions were chosen and a 2D image was acquired. With this method, at least 30–500 cells/sample were measured. 

To analyse the mixed cell suspensions shown in Figure 6, the corresponding samples were stained with APC-labelled α-CD3. A third bandpass filter (BP 675/67) was required to detect the emission of this marker. As the multiphoton microscope does not include a second laser source, the excitation wavelength was tuned to 1040 nm to evaluate the α-CD3 staining. A second image was then acquired at the same position at an excitation wavelength of 810 nm to excite natural autofluorescence as described above. Between these recordings, the focal plane was adjusted again to compensate for the different focal lengths at each wavelength. Both images were then combined for evaluation. This method is displayed in Supplementary Figure S2. 

Image analysis was performed with the open-source software Fiji (v1.52s, Wayne Rasband, National Institutes of Health, Bethesda, USA). For semi-automatic evaluation, a macro was implemented. In a first step, the image was thresholded in the NADH channel by the Otsu method and a binary mask was created upon manual confirmation by the user. Background noise was subtracted, and the regions of interest (ROIs) were determined based on the mask. Finally, intensity parameters of all channels (mean and median grey value and integrated density), as well as area of the cells, were measured. A mean value of the median grey value for all cells in one sample (including the three different positions) was normalized to the mean value of alive, unstimulated CD4+ T-cells, further described as NADH and FAD.

### 4.6. Preparation and Analysis of Mixed Cell Samples

To analyse the mixed cell samples shown in Figure 6, four different conditions of cell samples were prepared: CD4+ T cells alone, neutrophils alone, cell mixes of 50% CD4+ T cells + 50% neutrophils and cell mixes of 80% CD4+ T cells + 20% neutrophils. The lymphocyte marker α-CD3 (APC) was used as a reference to identify CD4+ T cells. APC and NADH signals of single cells were plotted as dot plots for flow cytometry and MPM. These dot plots were divided into four quadrants by defining groups of high and low NADH and APC values. In flow cytometry, these values were chosen by visual inspection of different populations in the FlowJo software; in MPM, a cut-off value of 67 for APC raw median grey values and a cut-off value of 778 for NADH raw median grey values were identified as best thresholds by ROC curves and Youden-Index.

### 4.7. Statistical Analysis

GraphPad Prism 8 (GraphPad software, San Diego, CA, USA) was used for statistical analysis. Normality was tested with Shapiro–Wilk test. As some groups of samples were not normally distributed, statistical tests for non-parametric distributions were used. Two independent groups were compared with a Mann–Whitney test, and for three or more independent groups a Kruskal–Wallis test with subsequent Dunn’s multiple comparisons test was performed. Boxplots represent median values, interquartile ranges, and the minimum and maximum values. Statistical significance is shown as follows: * *p* < 0.05, ** *p* < 0.01, *** *p* < 0.001, **** *p* < 0.0001.

## Data Availability

The data are available upon reasonable request to the corresponding authors.

## Supplementary Materials

The following are available online at https://www.mdpi.com/article/10.3390/ijms23042338/s1
