# Supplementary material for: Natural NADH and FAD Autofluorescence as Label-Free Biomarkers for Discriminating Subtypes and Functional States of Immune Cells
Source: Int J Mol Sci. 2022 Feb 20;23(4):2338. doi: 10.3390/ijms23042338 (PMC8880312; doi:10.3390/ijms23042338)
Supplement: Supplementary file 1 [file ijms-23-02338-s001.zip › ijms-1575714-supplementary.pdf]

International Journal of
Molecular Sciences

MDPI

Supplementary Material

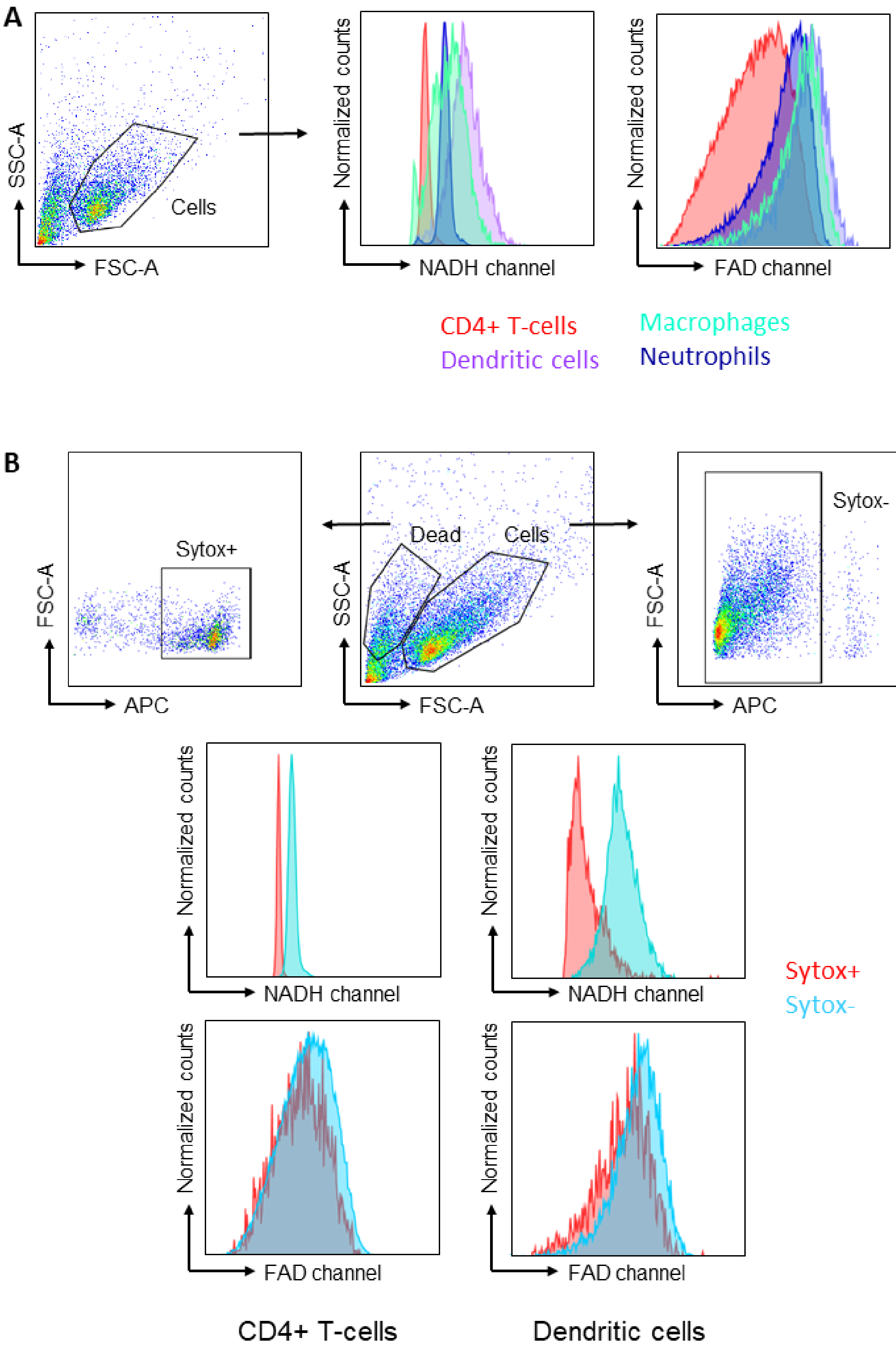

Figure S1. Gating strategy used for evaluation of flow cytometry data. (A) Overlay of NADH and FAD autofluorescence in different immune cell types. Cells were identified using forward and sideward scattering and autofluorescence was determined in the NADH and FAD channel. (B) Dead cells were identified using the cell death marker Sytox Red. Histograms show an overlay of NADH and FAD autofluorescence from dead and alive CD4+ T cells and dendritic cells.

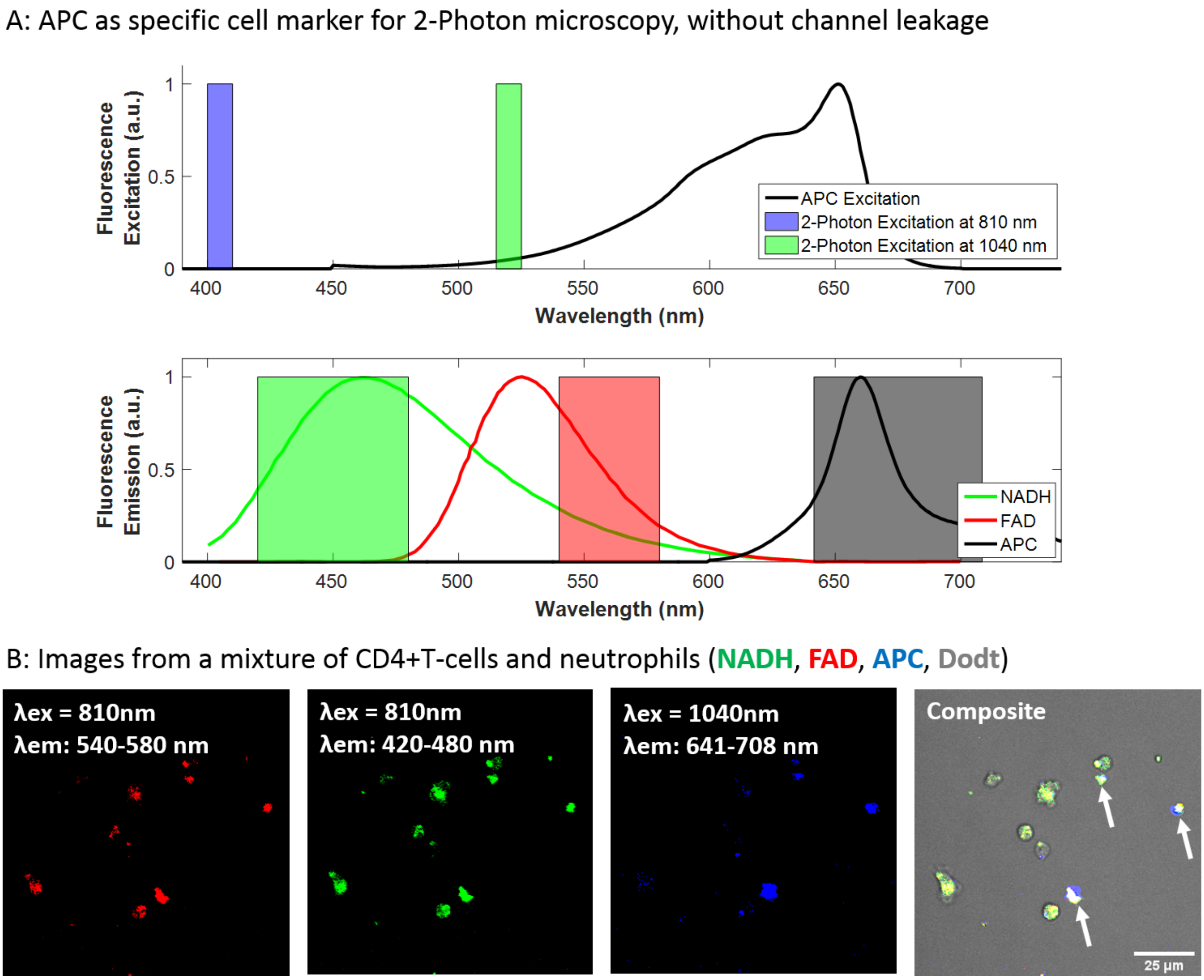

Figure S2. APC confirms possible differentiation of CD4+ T-cells and neutrophils based on NADH signals without leakage into autofluorescence channels (NADH and FAD). (A) Excitation and emission wavelengths of APC. (B) α-CD3-APC staining allows lymphocyte identification in a mixture of CD4+ T cells and neutrophils.
